# High *Plasmodium falciparum* longitudinal prevalence is associated with high multiclonality and reduced clinical malaria risk in a seasonal transmission area of Mali

**DOI:** 10.1371/journal.pone.0170948

**Published:** 2017-02-03

**Authors:** Yaw Adomako-Ankomah, Matthew S. Chenoweth, Katelyn Durfee, Saibou Doumbia, Drissa Konate, Mory Doumbouya, Abdoul S. Keita, Daria Nikolaeva, Gregory S. Tullo, Jennifer M. Anderson, Rick M. Fairhurst, Rachel Daniels, Sarah K. Volkman, Mahamadou Diakite, Kazutoyo Miura, Carole A. Long

**Affiliations:** 1 Laboratory of Malaria and Vector Research, National Institute of Allergy and Infectious Diseases, National Institutes of Health, Rockville, Maryland, United States of America; 2 Department of Immunology and Infectious Diseases, Harvard T.H. Chan School of Public Health, Boston, Massachusetts, United States of America; 3 Malaria Research and Training Center, Faculty of Medicine, Pharmacy, and Odontostomatology, University of Sciences, Techniques, and Technologies of Bamako, Bamako, Mali; 4 Department of Organismic and Evolutionary Biology, Harvard University, Cambridge, Massachusetts, United States of America; 5 Infectious Disease Program, The Broad Institute of MIT and Harvard, Cambridge, Massachusetts, United States of America; 6 School of Nursing and Health Sciences, Simmons College, Boston, Massachusetts, United States of America; Liverpool School of Tropical Medicine, UNITED KINGDOM

## Abstract

The effects of persistent *Plasmodium falciparum* (Pf) infection and multiclonality on subsequent risk of clinical malaria have been reported, but the relationship between these 2 parameters and their relative impacts on the clinical outcome of infection are not understood. A longitudinal cohort study was conducted in a seasonal and high-transmission area of Mali, in which 500 subjects aged 1–65 years were followed for 1 year. Blood samples were collected every 2 weeks, and incident malaria cases were diagnosed and treated. Pf infection in each individual at each time point was assessed by species-specific nested-PCR, and Pf longitudinal prevalence per person (PfLP, proportion of Pf-positive samples over 1 year) was calculated. Multiclonality of Pf infection was measured using a 24-SNP DNA barcoding assay at 4 time-points (two in wet season, and two in dry season) over one year. PfLP was positively correlated with multiclonality at each time point (all r≥0.36; all *P*≤0.011). When host factors (e.g., age, gender), PfLP, and multiclonality (at the beginning of the transmission season) were analyzed together, only increasing age and high PfLP were associated with reduced clinical malaria occurrence or reduced number of malaria episodes (for both outcomes, *P*<0.001 for age, and *P* = 0.005 for PfLP). When age, PfLP and baseline Pf positivity were analyzed together, the effect of high PfLP remained significant even after adjusting for the other two factors (*P* = 0.001 for malaria occurrence and *P*<0.001 for number of episodes). In addition to host age and baseline Pf positivity, both of which have been reported as important modifiers of clinical malaria risk, our results demonstrate that persistent parasite carriage, but not baseline multiclonality, is associated with reduced risk of clinical disease in this population. Our study emphasizes the importance of considering repeated parasite exposure in future studies that evaluate clinical malaria risk.

## Introduction

While major gains have been made in the control and elimination of malaria, this disease remains a major threat to global health with an estimated 438,000 global malaria-related deaths in 2015 [1]. Achieving further reductions in *Plasmodium falciparum* (Pf) transmission and infection burden in endemic areas will be facilitated by a better understanding of the true distribution of infections, and the host, parasite and vectorial factors that influence transmission and infection outcome. The application of updated, sensitive diagnostic tools to understanding how these factors interact and influence infection dynamics in different transmission settings will be essential for maximizing the impact of malaria control and elimination programs.

Studies investigating the dynamics of Pf prevalence have shown varying degrees of association between host factors, such as age, gender and red blood cell (RBC) polymorphisms, and Pf prevalence and clinical malaria in many epidemiological settings [2–9]. However, the majority of these studies utilized cross-sectional study designs [3, 5, 7–9] and in many cases focused on subpopulations in endemic areas based on age (adults or children) or infection outcome (symptomatic or asymptomatic). Such studies are unable to account for individual-level dynamics of Pf prevalence in all groups of people within the target population. Longitudinal analysis of individual-level Pf prevalence would provide more useful information about how different groups of people contribute to the parasite reservoir in an entire population during a full year of transmission.

One important feature of Pf infections, especially in areas of high transmission intensity, is that an individual could be concurrently infected with multiple, genetically-distinct clones of Pf. Multiclonality has been reported to influence the duration and outcome of infections [10–13], but these associations have been contradictory among studies. For example, severe malaria was significantly associated with high complexity of infection (COI) in Uganda [10], while a reduced risk of clinical malaria was associated with polygenomic *P*. *falciparum* infections in Papua New Guinea [14]. Furthermore, multiple studies have reported that new Pf infections following the clearance of previous polygenomic infections were less likely to progress to clinical malaria [12, 15, 16]. These studies notwithstanding, it remains unclear whether immunity to malaria is a consequence of exposure to the wide breadth of antigens simultaneously presented by a polygenomic infection or the result of extended parasite carriage, both of which are common features of Pf infections in high-transmission areas [12, 13, 17, 18].

In the present study, we followed a cohort of 500 individuals aged 1–65 years living in Kenieroba, Mali, where malaria transmission is highly seasonal [19, 20]. Using blood samples collected every 2 weeks for 1 year, we investigated the relationship between Pf longitudinal prevalence (PfLP) and multiclonality, and how these factors influence clinical malaria risk. Our results showed that PfLP, rather than multiclonality at baseline, was a better correlate of clinical disease in this population.

## Materials and methods

### Study site and population

This study was conducted in Kenieroba, a village of about 2,000 people located approximately 75 kilometers southwest of Bamako. In this high-transmission area of Mali, the annual rainy season occurs from June to December. The study cohort of 500 individuals aged 1–65 years was selected in proportion to the age distribution of the entire village population based on a village-wide census conducted in May 2012. Study participants were recruited based on their age and residency in Kenieroba for the full study year.

### Ethical approval

This study was approved by the Institutional Review Board of the National Institute of Allergy and Infectious Diseases and the Ethics Committee of the Faculty of Medicine, Pharmacy, and Odontostomatology, University of Bamako, and is registered with Clinicaltrials.gov, number NCT01829737. Written informed consent was obtained from study participants, or the parents or guardians of children aged <18 years.

### Sample collection and malaria case detection

Finger-prick blood samples, collected every 2 weeks from June 2013 to May 2014, were spotted on Whatman 3MM filter paper, and then dried and stored at room temperature until used. At the same time, 100 μL of whole blood were collected in 500 μL RNAprotect^®^ (Qiagen) and stored at –80°C. If participants had malaria symptoms (e.g., axillary temperature ≥37.5°C or history of fever in the past 2 weeks, headache, body aches, or malaise) at the time of a scheduled visit or self-referral, parasitological malaria diagnosis was performed by experienced study microscopists, and if confirmed, patients were referred to Kenieroba’s health center for standard-of-care antimalarial treatment. It should be noted that microscopy was not performed for the entire study. Parasite detection by Giemsa staining and subsequent microscopic analysis was only performed for the purpose of diagnosing clinical malaria in participants who presented with malaria symptoms as listed above, during the study period. The Kenieroba health center is the only health facility in the entire village and all study participants were encouraged to visit there when they developed a fever or other malaria symptoms. For the purpose of our analysis, clinical malaria was defined as axillary temperature ≥37.5°C and asexual parasite density >5000/μL by microscopy [12]. At the time of enrollment, ABO/Rh blood types, hemoglobin phenotype (e.g., AA, AS, or AC), and glucose-6-phosphate dehydrogenase (G6PD) deficiency and α-thalassemia genotypes were determined for each participant, as previously described [21].

### PCR detection of *Plasmodium spp*. infections

Molecular diagnostic detection of *P*. *falciparum*, *P*. *malariae*, *P*. *vivax and P*. *ovale* infections was performed using species-specific nested-PCR [22], with dried blood spots on filter paper directly without prior genomic DNA extraction, as described previously [23]. Briefly, for each sample, a 1mm circular punch of dried blood spot was added directly to the primary reaction of a nested-PCR targeting the plasmodial 18S rDNA. Species-specific primers were used in separate secondary reactions for diagnostic detection of the four *Plasmodium* spp., [22]. The nested-PCRs and their thermocycler profiles are outlined in S1 and S2 Tables. PCR products were analyzed by capillary electrophoresis on the LabChip GX HT with a HT DNA 5K LabChip, according to the manufacturer’s protocol (Caliper Life Sciences).

### Single-nucleotide polymorphism DNA barcoding

Pf-positive samples (by nested-PCR) from June 2013 (Jun-V1), November 2013 (Nov-V1), February 2014 (Feb-V1), and April 2014 (Apr-V1) were selected for DNA barcoding [24]. For each blood sample stored in RNAprotect^®^, parasite genomic DNA (gDNA) was extracted as follows: gDNA samples were eluted from a gDNA eliminator column using the QIAmp 96 Blood Kit (Qiagen) protocol, as modified by Wampfler et al [25]. Samples were eluted twice following 30-minute incubations in pre-warmed (40°C) TE buffer. Extracted gDNA samples were stored at –20°C until used. For single-nucleotide polymorphism (SNP) barcoding, extracted gDNA samples were subjected to a previously described pre-amplification protocol [26]. Each pre-amplified gDNA sample was used as template in a bi-allelic 24-SNP barcode assay. Details of this assay, including primer and probe sequences, have been described [24]. Polymorphic or monomorphic calls for each SNP assay were subsequently used to generate a molecular barcode for each Pf infection. S3 Table shows the breakdown of the SNP barcode assay data by the 24 reactions (S3 Table).

Complexity of infection (COI) was determined for each DNA sample using the COIL program as described previously [27], with a minor modification. In brief, the program utilizes binomial distribution to estimate COI from bi-allelic SNP genotyping data based on: 1) the population minor allele frequency (MAF) for each SNP locus, and 2) the number of polymorphic genotypes in each 24-SNP barcode [27]. COIL was run with default settings [28] (i.e., genotyping error rate of 5% and a uniform prior) with the exception of the MAFs, which were manually calculated at each SNP position. From the 24-SNP barcode, COIL only has the power to distinguish between COI levels of 1, 2, and ≥3.

### Statistical and data analysis

At each time-point, population-level Pf prevalence was calculated as the proportion (%) of individuals who were Pf-positive by nested-PCR among the total number of individuals who provided blood samples, regardless of whether they received antimalarial treatment before sample collection. *P*. *falciparum* longitudinal prevalence (PfLP) was calculated for each individual as the proportion (%) of the number of Pf-positive samples among the total number of samples collected over 1 year. Only data from individuals with available samples from at least two-thirds of the 22 time-points (i.e., ≥15 samples; 430/500 individuals enrolled) were analyzed. At each of the 4 selected time-points, Polymorphic Proportion (PmP) was calculated for each Pf-positive individual (sample), as the proportion (%) of polymorphic reactions among the total number of successful reactions (reactions that yielded monomorphic or polymorphic nucleotide calls) from the 24 reactions that make up the 24-SNP barcoded assay. Only data from samples with ≥13 successful assays (Jun-V1, 96%; Nov-V1, 82%; Feb-V1, 88%; and Apr-V1, 98%) were analyzed.

Age- and gender-stratified PfLP were analyzed using Kruskal-Wallis test followed by Dunn’s multiple comparison test and Mann-Whitney test, respectively. COI and PmP distributions were analyzed between time-points using Chi-squared test and Kruskal-Wallis test, respectively. Associations between PfLP and PmP were analyzed using Spearman rank correlation test. A multiple linear regression analysis was performed to evaluate the association between response and multiple explanatory variables (e.g., effects of host factors on PfLP). For clinical data, a logistic regression model was used to analyze the risk of experiencing clinical malaria over 1 year, and Poisson and Cox's proportional hazard regression models were used to analyze the number of malaria episodes and time to first clinical malaria episode, respectively.

## Results

### Malaria incidence and cross-sectional prevalence of *P*. *falciparum* infection in Kenieroba

**Fig 1:**
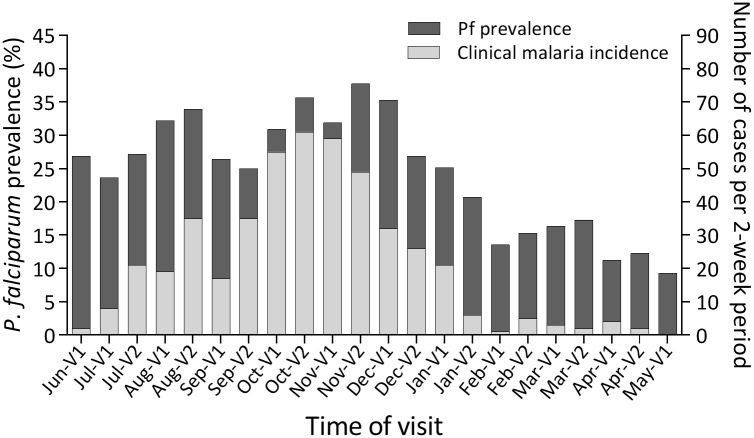
Cross-sectional prevalence of *P*. *falciparum* (Pf) infection and 2-week incidence of clinical malaria from June 2013 to May 2014. Left y-axis: Pf prevalence measured by nested-PCR; dark gray bars represent Pf prevalence. Right y-axis: number of clinical malaria cases recorded during each 2-week period preceding a blood sampling visit; light gray bars represent 2-week incidence. The time of visit is indicated on the x-axis, with V1 and V2 indicating the first and second blood sampling visits, respectively, for the months indicated.

The 500-subject cohort, representing approximately a quarter of the population in Kenieroba, was drawn to reflect the age distribution of the entire village population. The demographic characteristics of the cohort are summarized in Table 1. Fig 1 shows the number of clinical malaria cases recorded among study participants for the 2-week period preceding each round of blood sample collection. A total of 469 cases were recorded among 232 participants over 1 year. Eighty-six percent of these cases were recorded in children aged ≤10 years. Occurrence and number of malaria episodes in each age category are shown in Table 2. Cross-sectional prevalence of Pf infection, measured by nested-PCR, was highest in November 2013 (Nov-V2, Fig 1) and lowest in May 2014 (May-V1). Eighty-five percent (427/500) of individuals had at least 1 Pf-positive blood sample over 1 year. The prevalence of other *Plasmodium* spp. in this area were also assessed at 2 wet season (Jun-V1 and Nov-V1) and 2 dry season (Feb-V1 and Apr-V1) time-points using species-specific nested-PCR. No *P*. *ovale* or *P*. *vivax* infections were detected while *P*. *malariae* infections were observed at all 4 time-points but at low levels (Jun-V1, 5%; Nov-V1, 2%; Feb-V1, 4%; and Apr-V1, 3% within the entire cohort). Therefore, further analysis was performed exclusively on *P*. *falciparum* infections.

**Table 1:** Demographic characteristics of the cohort.

Age ^b^ (years)	Gender		ABO^a^				Rh^a^		Hb Type^a^					G6PD deficiency^a^			α-thalassemia^a^		
	Male	Female	A	AB	B	O	+	-	AA	AC	AS	CC	SC	A-	A+	A+/-	HE	HO	WT
1–2	22	18	12	5	6	16	38	1	30	4	5	0	0	4	32	3	15	1	22
3–4	21	33	15	11	12	16	53	0	39	7	7	0	1	1	45	7	11	2	40
5–6	19	29	17	1	13	17	42	6	33	6	9	0	0	2	40	6	21	0	27
7–8	33	28	22	5	12	22	58	3	47	5	8	0	0	5	48	6	21	1	37
9–10	31	27	18	4	18	18	55	3	38	7	11	1	1	2	52	4	12	1	45
11–12	24	27	17	3	14	17	46	5	42	5	3	0	1	1	45	4	16	1	34
13–16	33	24	10	4	22	21	53	4	44	3	9	0	1	2	49	6	14	1	41
17–30	9	39	15	2	10	21	47	1	38	3	4	0	1	3	38	4	20	1	23
31–40	5	33	11	3	14	10	38	0	28	3	6	0	0	2	31	4	19	0	18
>40	19	26	12	4	11	18	44	1	38	0	7	0	0	2	33	9	13	3	27
Total	216	284	149	42	132	176	474	24	377	43	69	1	5	24	413	53	162	11	314

^a^ Individuals with no available data were excluded

^b^ Subjects were divided into 10 age groups based on their age and number of subjects so that each group contained around 10% of the cohort.

**Table 2:** Malaria episodes in each age category.

Age	N	Occurrence of malaria^a^	Number of malaria episodes^b^
1–2	40	67.5	1.7 (1.5)
3–4	54	79.6	1.7 (1.5)
5–6	48	83.3	2.1 (1.5)
7–8	61	77.0	1.6 (1.3)
9–10	58	53.4	0.8 (0.9)
11–12	51	45.1	0.8 (1.1)
13–16	57	19.3	0.2 (0.6)
17–30	48	6.3	0.1 (0.3)
31–40	38	10.5	0.1 (0.3)
>40	45	6.7	0.1 (0.4)
Total	500	46.4	0.9 (1.3)

^a^ Proportion (%) of individuals who experienced any malaria episodes

^b^ Arithmetic mean (standard deviation)

### *P*. *falciparum* longitudinal prevalence in the cohort correlates with age and gender

**Fig 2:**
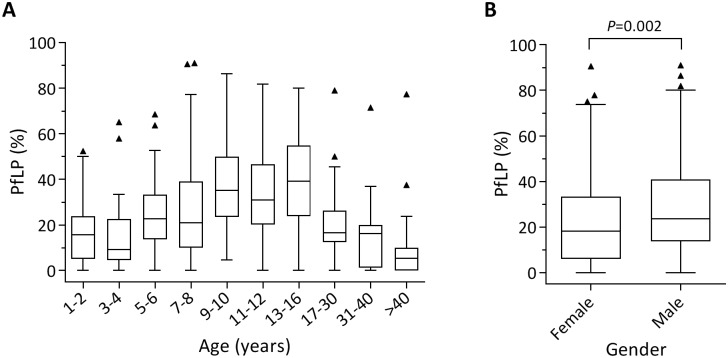
*P*. *falciparum* longitudinal prevalence (PfLP) stratified by age (A) and gender (B). Subjects were divided into 10 age groups (A) based on their age and number of subjects so that each group contained around 10% of the cohort (38 to 61 individuals per group, Table 1). Box-and-whisker plots show median, interquartile range (IQR), 1.5x IQR, and outliers (triangles). Differences between age groups were analyzed by Kruskal-Wallis test followed by Dunn’s multiple comparison test (see S4 Table for adjusted *P* values). PfLP differed significantly by gender (*P* = 0.0024, Mann-Whitney test).

We calculated the PfLP for each individual, and determined how it was affected by age, gender, and RBC polymorphisms including ABO/Rh types, Hb phenotype (e.g. Hb AA, Hb AS), and G6PD deficiency and α-thalassemia genotypes. When host factors were evaluated individually, or together using multiple linear regression analysis (data not shown), only age and gender were significantly associated with PfLP. PfLP increased with age until 9–16 years, after which it declined with increasing age (Fig 2A). Individuals aged 9–16 years had significantly higher PfLP than those aged >17 years or 1–4 years (Dunn’s multiple comparison test; S4 Table). PfLP was also significantly higher in males than females (Fig 2B; Mann-Whitney test, *P* = 0.002), even after adjusting for age (*P* = 0.010). Thus, host age and gender were significantly associated with PfLP in this population.

### High proportion of multiclonal *P*. *falciparum* infections in the cohort

**Fig 3:**
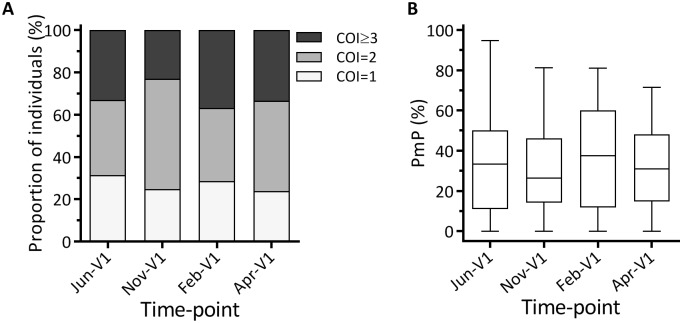
Multiclonality of *P*. *falciparum* infection at 4 time-points. (A) Distribution of complexity of infection (COI) values estimated by COIL. The proportions of individuals with COI = 1 (white), COI = 2 (light gray), and COI≥3 (dark gray) at each time-point are indicated. (B) Distribution of polymorphic proportion (PmP) values calculated from polymorphic genotypes in the 24-SNP DNA barcode. Box-and-whisker plots show median, interquartile range (IQR), and 1.5x IQR. There were no significant differences in the COI and PmP distributions among the 4 time-points as determined by Chi-squared and Kruskal-Wallis tests, respectively.

We measured the multiclonality of Pf infections at the same wet and dry season time-points at which the prevalence of other *Plasmodium* spp. were measured (Jun-V1, Nov-V1, Feb-V1 and Apr-V1). For this purpose, we used the 24-SNP DNA barcoding assay developed by Daniels et al [24]. From the barcode data for each sample, COI was estimated with the COIL program [27], and PmP was calculated based on the number of polymorphic assays. As expected, individuals with high COI also had high PmP (S1 Fig). COI estimates revealed that ≥70% of Pf infections were polygenomic (COI≥2) at all 4 time-points, and there was no difference in the COI distributions among the 4 time-points (Fig 3A; Chi-squared test, *P* = 0.192). Consistent with this analysis, there was no significant difference in the PmP distributions among the 4 time-points (Fig 3B; Kruskal-Wallis test; *P* = 0.557). In addition, we examined the geographical distributions of Pf positivity, COI, and PmP within the study area at all 4 time-points and found no clustering of infections representing significant hotspots (data not shown).

### Multiclonality and longitudinal prevalence are positively correlated in the cohort

**Fig 4:**
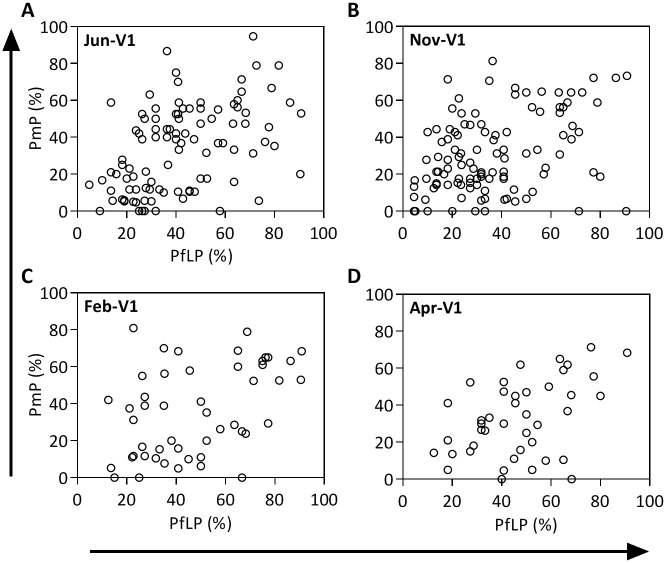
Correlation between polymorphic proportion (PmP) and *P*. *falciparum* longitudinal prevalence (PfLP). PmP positively correlates with PfLP at all 4 time-points: (A) Jun-V1 (r = 0.47, 95% confidence interval = 0.30–0.61, *P*<0.001, Spearman rank correlation test); (B) Nov-V1 (r = 0.36, 0.18–0.52, *P*<0.001); (C) Feb-V1 (r = 0.42, 0.15–0.63, *P* = 0.003); and (D) Apr-V1 (r = 0.39, 0.09–0.63, *P* = 0.011)

The relationship between PfLP and multiclonality was analyzed using PmP instead of COI because PmP yielded a higher resolution of multiclonality than the 3-level COI, which is the limit of COIL analysis (Fig 4). PmP and PfLP showed significant positive correlations at all 4 time-points although their correlation coefficients were relatively modest (Spearman rank correlation coefficients were between 0.36 and 0.47; all *P* = <0.011). Since there was a significant correlation between PmP and PfLP, similar age and gender effects on PmP were also observed, as expected (data not shown). Furthermore, the associations between PmP and PfLP remained significant after adjusting for age and gender (all *P*<0.007). Thus, these results indicate a positive relationship between multiclonality and the frequency of Pf positivity in this population.

### *P*. *falciparum* longitudinal prevalence, but not baseline multiclonality, was correlated with clinical malaria risk

Using the Jun-V1 time-point as a baseline, we analyzed the impact of host factors, PfLP, and PmP on 3 measures of malaria risk over 1 year: 1) occurrence of malaria, 2) number of malaria episodes, and 3) time to first malaria episode (Table 3). For this analysis, we included all Pf-positive individuals (no PmP data for Pf-negative individuals) at baseline who had data for PmP and PfLP (n = 103), and the individuals were categorized into two subgroups based on their PfLP or PmP levels (i.e., high = above 75^th^ percentile, and low = below 75^th^ percentile). Since ABO/Rh types, Hb phenotype, G6PD deficiency, and α-thalassemia showed no significant impact on the clinical malaria readouts, those factors were excluded in the final analysis (data not shown). Logistic regression analysis showed that increasing age (*P*<0.001) and high PfLP (*P* = 0.005) were associated with reduced risk of malaria occurrence. Similarly, Poisson regression analysis showed that increasing age (*P*<0.001) and high PfLP (*P* = 0.005) were associated with decreasing number of malaria episodes. PmP and gender were not significantly associated with malaria occurrence or number of malaria episodes, and none of the factors were associated with time to first malaria episode. To further evaluate the impact of PfLP on clinical malaria risk, we included baseline Pf positivity as a covariate in the analysis (Table 4). This analysis included 463 individuals (124 were Pf-positive) who had data for Pf infection status at baseline and for PfLP. After adjusting for baseline Pf positivity, high PfLP was still significantly associated with malaria occurrence (*P* = 0.001) and number of malaria episodes (*P*<0.001). Thus, in addition to host age, high PfLP was associated with reduced risk of clinical malaria.

**Table 3:** Results of multivariate regression analysis of clinical malaria risk^a^.

Explanatory variables	Occurrence of malaria		Number of malaria episodes		Time to first malaria episode	
	OR^b^ (95% CI^c^)	*P*	IRR^d^ (95% CI)	*P*	HR^e^ (95% CI)	*P*
Age	0.81 (0.71–0.90)	**<0.001**	0.61 (0.48–0.76)	**<0.001**	0.96 (0.87–1.02)	0.227
Gender	0.61 (0.23–1.59)	0.317	0.77 (0.34–1.74)	0.526	0.90 (0.49–1.67)	0.735
PfLP_75_^f^	0.24 (0.08–0.65)	**0.005**	0.28 (0.11–0.68)	**0.005**	0.68 (0.34–1.37)	0.283
PmP_75_^g^	1.65 (0.55–5.25)	0.377	1.01 (0.37–2.77)	0.983	1.66 (0.67–3.86)	0.269

^a^ Data from 103 individuals were analyzed

^b^ OR, odds ratio

^c^ CI, confidence interval

^d^ IRR, incidence rate ratio

^e^ HR, hazard ratio

^f^ Individuals were divided into two subgroups based on their PfLP levels (more than 75 percentile of whole population or not)

^g^ Individuals were divided into two subgroups based on their PmP levels at Jun-V1 (more than 75 percentile of whole population or not)

**Table 4:** Results of multivariate regression analysis of clinical malaria risk, including baseline Pf positivity^a^.

Explanatory variables	Occurrence of malaria		Number of malaria episodes		Time to first malaria episode	
	OR^b^ (95%CI^c^)	*P*	IRR^d^ (95% CI)	*P*	HR^e^ (95%CI)	*P*
Age	0.88 (0.86–0.91)	**<0.001**	0.78 (0.73–0.82)	**<0.001**	1.01 (0.99–1.03)	0.410
PfLP_75_^f^	0.35 (0.20–0.60)	**0.001**	0.37 (0.22–0.61)	**<0.001**	1.07 (0.74–1.53)	0.692
Pf positivity^g^	0.66 (0.38–1.16)	0.146	0.73 (0.45–1.18)	0.205	0.86 (0.61–1.20)	0.386

^a^ Data from 463 individuals were analyzed

^b^ OR, odds ratio

^c^ CI, confidence interval

^d^ IRR, incidence rate ratio

^e^ HR, hazard ratio

^f^ Individuals were divided into two subgroups based on their PfLP levels (more than 75 percentile of whole population or not)

^g^ Individuals were divided into two subgroups based on their Pf positivity at baseline (Jun-V1)

## Discussion

In a region of intense seasonal malaria transmission in Mali, our cohort study determined that *P*. *falciparum* longitudinal prevalence was positively correlated with multiclonality (Fig 4). Interestingly, we found that longitudinal prevalence (and age), but not multiclonality at baseline, was associated with reduced risk of clinical malaria over 1 year (Table 3). The effect of PfLP remained significant even after adjusting for baseline Pf positivity (Table 4). Since PfLP is a parameter of Pf carriage that measures the number of times an individual is Pf-positive over a time-period, these results imply that the frequency or duration of exposure to Pf, rather than the genomic diversity of the infection, may be a better indicator of clinical malaria risk. These results suggest that individuals with higher PfLP are more likely to have acquired effective protective immunity against clinical malaria. While several studies have assessed the impacts of baseline Pf positivity [12, 29, 30] and multiclonality on clinical malaria risk, they have not analyzed PfLP as an endpoint. Indeed, when the regression analyses were performed without the PfLP factor, Pf positivity at baseline was significantly associated with malaria occurrence and number of malaria episodes after adjusting for age (individuals who were Pf positive at baseline had less chance to experience malaria episodes, data not shown). Since PfLP, PmP and Pf positivity were all correlated (e.g., individuals with higher PfLP are likely to show higher PmP and higher chance of Pf positivity at baseline), it is necessary to analyze these multiple factors together to determine the true correlations. In our analysis, we included data-points regardless of whether individuals were treated for clinical malaria, since excluding data from individuals who had received treatment within 2 weeks prior to blood sampling had no impact on the main conclusions described above. Similarly, using different parasitemia cut-off values (i.e., any parasitemia, parasite density above 1000 or 2500/μL, instead of the 5000/μL used in this manuscript) to define clinical malaria cases did not influence the main conclusions.

The effects of host age on cross-sectional Pf prevalence have been previously reported; in Senegal [6] and Tanzania [31], for example, Pf prevalence was consistently lower in younger children and adults relative to the intervening age groups. Consistent with these findings, our analysis of PfLP revealed a significant effect of host age on PfLP, which peaked in children aged 9–16 years. Although we did not assess the year-to-year stability of the age-PfLP relationship in our study population, age-associated effects on Pf prevalence have been observed in several places where decreasing transmission intensity was associated with a shift in peak Pf prevalence towards older age groups [6, 32, 33]. It will therefore be necessary to re-evaluate these dynamics as local transmission intensity changes. Furthermore, while our study cannot explain the higher PfLP in males in this population, it is possible that the observed gender effect on PfLP results from a combination of socio-cultural and behavioral factors that leads to differential exposure to mosquito bites. Traditional gender roles and sleeping behaviors, including disparity in bed net usage, have been considered to partly underlie gender effects on cross-sectional Pf prevalence in other studies [5, 34, 35]. Gender-based differences in immune responses to Pf infection have also been proposed as a possible explanation [36] for these differences.

Genotyping of polymorphic genes, such as *MSP1* and *MSP2*, is a widely-used method to detect multiclonal Pf infections. Such assays, using markers with a high number of distinguishable alleles, have the power to resolve complexity of infections but only at a single or few loci. For this study we chose to use a 24-SNP DNA barcoding assay since it utilizes 24 independent markers and is therefore expected to have higher sensitivity to differentiate between monogenomic and polygenomic infections among Pf-positive individuals. Since this barcoding technique has only been used for monitoring parasite population dynamics thus far [24, 37–39], this study expands its applications. Our preliminary data showed that SNP barcoding detected 20% more polygenomic infections than *MSP2* genotyping at the Nov-V1 time-point (data not shown). Furthermore, >70% of Pf infections in our cohort at all 4 time-points were found to be polygenomic, which is about 25% higher than that measured by *MSP2* genotyping in Kambila, Mali, where transmission dynamics are similar to those in Kenieroba [12]. In addition to the high prevalence of multiclonality, the distributions of COI and PmP were also similar among the 4 time-points, although there was a clear seasonal effect on Pf prevalence (Fig 1). These results suggest that the endemicity during the dry season (~10% or more individuals were Pf positive) might still be too high to lead to change in COI and PmP under these transmission conditions. There are two caveats to the interpretation of these data. The absence of seasonal effect on COI and PmP at the population level does not necessarily mean that multiclonality within individuals is stable throughout the year. In addition, the current study was not designed to assess the duration of carriage for individual clones of parasites *in vivo*. Therefore, we cannot address whether there were any differences in the duration of infection by individual clones between wet and dry seasons.

Our analysis revealed a significant correlation between PmP and PfLP, highlighting the importance of multiclonality as an indicator of parasite carriage, particularly in high-transmission areas where the prevalence of polygenomic infections may exceed 70%. However, the correlation coefficients were ≤0.47, meaning that one measure can explain ≤47% of variance in another measure. As a result, PfLP was significantly associated with the occurrence of clinical malaria and the number of episodes over 1 year, but PmP at baseline was not. These findings are consistent with previous reports and support a model of protection against clinical malaria resulting from persisting or repeated parasite carriage, but not necessarily the number of clones at any specific time-point [12, 15, 16, 40]. Interestingly, there was no association between any of the explanatory variables tested and time to first malaria episode, which has been used as an additional measure of risk in some studies [12, 15, 16, 41]. While time to first episode is a relevant measure of risk in drug clearance studies, there is no consensus on which of the three measures (i.e., occurrence, number of episodes, or time to first episode) is the best indicator of immunity against clinical malaria in non-interventional studies such as the current study, especially in areas where asymptomatic infections are common. And since the age factor also showed no significant correlation, time to first malaria episode might not be a suitable indicator in this study setting.

Our study had a number of limitations. We did not measure parasite density as an endpoint and therefore we are unable to assess correlations between parasite density and host factors as well as parasite factors such as COI in this population. Moreover, future studies will need to investigate the impacts of other extrinsic determinants of transmission and parasite carriage such as rainfall, temperature, and usage of transmission-control tools (e.g., bed nets and indoor residual spraying which may introduce household-level variations) on PfLP. Our study was limited to 1 year and will need to be extended to determine the stability of the age and gender effects on PfLP with changing transmission dynamics. More participants may also be needed to properly evaluate the effects of several underrepresented host factors (e.g., RBC polymorphisms) on PfLP and clinical malaria risk. In addition, *P*. *malariae* which was the only other *Plasmodium* spp. detected in our cohort occurred only rarely, and it has been reported that other less commonly detected species such as *P*. *ovale wallikeri* are missed by the detection method used in this study [42]. Therefore the effect of *Plasmodium* spp. co-infections on persistent carriage and malaria risk should be evaluated in a different setting with more targeted methodologies.

In conclusion, our study demonstrates that in the context of high seasonal malaria transmission, multiclonality (PmP) was positively associated with persistent Pf positivity (PfLP), but only persistent Pf carriage was significantly associated with clinical malaria risk when age and baseline Pf positivity were adjusted for in this population. Our results suggest that individuals with persistent Pf carriage are likely to have acquired a more effective protective immunity against clinical malaria. Our study emphasizes the importance of considering repeated parasite exposure, in addition to Pf positivity at baseline, in future studies that evaluate clinical malaria risk.

## Supporting information

S1 Fig Distribution of polymorphic proportion (PmP) values among 3 COIL-estimated complexity of infection (COI) levels. At all 4 time-points (*A*-*D*), individuals with high COI also had high PmP (Kruskal-Wallis test, all *P*<0.001).
(PDF)

S1 Table Nested PCRs to Detect *P*. *falciparum* in Dried Blood Spots. (DOCX)

S2 Table Thermocycler Profiles of Nested PCRs to Detect *P*. *falciparum* in Dried Blood Spots. (DOCX)

S3 Table Results of 24-SNP barcode assay at 4 time-points broken down by the 24 loci. (XLSX)

S4 Table Adjusted *P* Values* from Dunn’s Multiple Comparison Test for Age-stratified *P*. *falciparum* Longitudinal Prevalence (PfLP) Data. (DOCX)

## References

[1] WHO. World Health Organization. World Malaria Report. 2015.

[2] Baliraine FN, Afrane YA, Amenya DA, Bonizzoni M, Menge DM, Zhou G, et al High prevalence of asymptomatic *Plasmodium falciparum* infections in a highland area of western Kenya: a cohort study. J Infect Dis. 2009;200(1):66–74. 10.1086/599317 19476434 PMC2689925

[3] Kaisar MM, Supali T, Wiria AE, Hamid F, Wammes LJ, Sartono E, et al Epidemiology of *Plasmodium* infections in Flores Island, Indonesia using real-time PCR. Malar J. 2013;12:169 10.1186/1475-2875-12-169 23706132 PMC3679745

[4] Pinkevych M, Petravic J, Chelimo K, Vulule J, Kazura JW, Moormann AM, et al Decreased growth rate of *P*. *falciparum* blood stage parasitemia with age in a holoendemic population. J Infect Dis. 2014;209(7):1136–43. 10.1093/infdis/jit613 24265441 PMC3952668

[5] Jenkins R, Omollo R, Ongecha M, Sifuna P, Othieno C, Ongeri L, et al Prevalence of malaria parasites in adults and its determinants in malaria endemic area of Kisumu County, Kenya. Malar J. 2015;14:263 10.1186/s12936-015-0781-5 26152272 PMC4495611

[6] Roucher C, Rogier C, Dieye-Ba F, Sokhna C, Tall A, Trape JF. Changing malaria epidemiology and diagnostic criteria for *Plasmodium falciparum* clinical malaria. PLoS One. 2012;7(9):e46188 10.1371/journal.pone.0046188 23029433 PMC3460864

[7] Betuela I, Maraga S, Hetzel MW, Tandrapah T, Sie A, Yala S, et al Epidemiology of malaria in the Papua New Guinean highlands. Trop Med Int Health. 2012;17(10):1181–91. 10.1111/j.1365-3156.2012.03062.x 22925472

[8] Donnelly B, Berrang-Ford L, Labbe J, Twesigomwe S, Lwasa S, Namanya DB, et al *Plasmodium falciparum* malaria parasitaemia among indigenous Batwa and non-indigenous communities of Kanungu district, Uganda. Malar J. 2016;15(1):254 10.1186/s12936-016-1299-1 27146298 PMC4855715

[9] Golassa L, Enweji N, Erko B, Aseffa A, Swedberg G. Detection of a substantial number of sub-microscopic *Plasmodium falciparum* infections by polymerase chain reaction: a potential threat to malaria control and diagnosis in Ethiopia. Malar J. 2013;12:352 10.1186/1475-2875-12-352 24090230 PMC3850638

[10] Kiwuwa MS, Ribacke U, Moll K, Byarugaba J, Lundblom K, Farnert A, et al Genetic diversity of *Plasmodium falciparum* infections in mild and severe malaria of children from Kampala, Uganda. Parasitol Res. 2013;112(4):1691–700. 10.1007/s00436-013-3325-3 23408340 PMC3597336

[11] Babiker HA, Gadalla AA, Ranford-Cartwright LC. The role of asymptomatic *P*. *falciparum* parasitaemia in the evolution of antimalarial drug resistance in areas of seasonal transmission. Drug Resist Updat. 2013;16(1–2):1–9. 10.1016/j.drup.2013.02.001 23510592

[12] Sonden K, Doumbo S, Hammar U, Vafa Homann M, Ongoiba A, Traore B, et al Asymptomatic Multiclonal *Plasmodium falciparum* Infections Carried Through the Dry Season Predict Protection Against Subsequent Clinical Malaria. J Infect Dis. 2015;212(4):608–16. 10.1093/infdis/jiv088 25712968 PMC4539894

[13] Nassir E, Abdel-Muhsin AM, Suliaman S, Kenyon F, Kheir A, Geha H, et al Impact of genetic complexity on longevity and gametocytogenesis of *Plasmodium falciparum* during the dry and transmission-free season of eastern Sudan. Int J Parasitol. 2005;35(1):49–55. 10.1016/j.ijpara.2004.10.014 15619515

[14] al-Yaman F, Genton B, Reeder JC, Anders RF, Smith T, Alpers MP. Reduced risk of clinical malaria in children infected with multiple clones of *Plasmodium falciparum* in a highly endemic area: a prospective community study. Trans R Soc Trop Med Hyg. 1997;91(5):602–5. 9463681 10.1016/s0035-9203(97)90046-8

[15] Liljander A, Bejon P, Mwacharo J, Kai O, Ogada E, Peshu N, et al Clearance of asymptomatic *P*. *falciparum* Infections Interacts with the number of clones to predict the risk of subsequent malaria in Kenyan children. PLoS One. 2011;6(2):e16940 10.1371/journal.pone.0016940 21383984 PMC3044709

[16] Farnert A, Williams TN, Mwangi TW, Ehlin A, Fegan G, Macharia A, et al Transmission-dependent tolerance to multiclonal *Plasmodium falciparum* infection. J Infect Dis. 2009;200(7):1166–75. 10.1086/605652 19702508 PMC2741682

[17] Roper C, Richardson W, Elhassan IM, Giha H, Hviid L, Satti GM, et al Seasonal changes in the *Plasmodium falciparum* population in individuals and their relationship to clinical malaria: a longitudinal study in a Sudanese village. Parasitology. 1998;116(Pt 6):501–10. 9651932 10.1017/s0031182098002650

[18] Nsango SE, Abate L, Thoma M, Pompon J, Fraiture M, Rademacher A, et al Genetic clonality of *Plasmodium falciparum* affects the outcome of infection in Anopheles gambiae. Int J Parasitol. 2012;42(6):589–95. 10.1016/j.ijpara.2012.03.008 22554991

[19] Miura K, Diakite M, Diouf A, Doumbia S, Konate D, Keita AS, et al Relationship between malaria incidence and IgG levels to *Plasmodium falciparum* merozoite antigens in Malian children: impact of hemoglobins S and C. PLoS One. 2013;8(3):e60182 10.1371/journal.pone.0060182 23555917 PMC3610890

[20] Lopera-Mesa TM, Doumbia S, Konate D, Anderson JM, Doumbouya M, Keita AS, et al Effect of red blood cell variants on childhood malaria in Mali: a prospective cohort study. Lancet Haematol. 2015;2(4):e140–9. 10.1016/S2352-3026(15)00043-5 26687956 PMC4418020

[21] Crompton PD, Traore B, Kayentao K, Doumbo S, Ongoiba A, Diakite SA, et al Sickle cell trait is associated with a delayed onset of malaria: implications for time-to-event analysis in clinical studies of malaria. J Infect Dis. 2008;198(9):1265–75. 10.1086/592224 18752444 PMC2574881

[22] Snounou G, Viriyakosol S, Zhu XP, Jarra W, Pinheiro L, do Rosario VE, et al High sensitivity of detection of human malaria parasites by the use of nested polymerase chain reaction. Mol Biochem Parasitol. 1993;61(2):315–20. 8264734 10.1016/0166-6851(93)90077-b

[23] Tran TM, Li S, Doumbo S, Doumtabe D, Huang CY, Dia S, et al An intensive longitudinal cohort study of Malian children and adults reveals no evidence of acquired immunity to *Plasmodium falciparum* infection. Clin Infect Dis. 2013;57(1):40–7. 10.1093/cid/cit174 23487390 PMC3669526

[24] Daniels R, Volkman SK, Milner DA, Mahesh N, Neafsey DE, Park DJ, et al A general SNP-based molecular barcode for *Plasmodium falciparum* identification and tracking. Malar J. 2008;7:223 10.1186/1475-2875-7-223 18959790 PMC2584654

[25] Wampfler R, Timinao L, Beck HP, Soulama I, Tiono AB, Siba P, et al Novel genotyping tools for investigating transmission dynamics of *Plasmodium falciparum*. J Infect Dis. 2014;210(8):1188–97. 10.1093/infdis/jiu236 24771862 PMC4271069

[26] Mharakurwa S, Daniels R, Scott A, Wirth DF, Thuma P, Volkman SK. Pre-amplification methods for tracking low-grade *Plasmodium falciparum* populations during scaled-up interventions in Southern Zambia. Malar J. 2014;13:89 10.1186/1475-2875-13-89 24618119 PMC4007587

[27] Galinsky K, Valim C, Salmier A, de Thoisy B, Musset L, Legrand E, et al COIL: a methodology for evaluating malarial complexity of infection using likelihood from single nucleotide polymorphism data. Malar J. 2015;14:4 10.1186/1475-2875-14-4 25599890 PMC4417311

[28] COIL_Estimator. GitHub COIL2 repository. https://github.com/COIL2/COIL2.

[29] Le Port A, Cot M, Etard JF, Gaye O, Migot-Nabias F, Garcia A. Relation between *Plasmodium falciparum* asymptomatic infection and malaria attacks in a cohort of Senegalese children. Malar J. 2008;7:193 10.1186/1475-2875-7-193 18823542 PMC2567330

[30] Males S, Gaye O, Garcia A. Long-term asymptomatic carriage of *Plasmodium falciparum* protects from malaria attacks: a prospective study among Senegalese children. Clin Infect Dis. 2008;46(4):516–22. 10.1086/526529 18199040

[31] Hofmann N, Mwingira F, Shekalaghe S, Robinson LJ, Mueller I, Felger I. Ultra-sensitive detection of *Plasmodium falciparum* by amplification of multi-copy subtelomeric targets. PLoS Med. 2015;12(3):e1001788 10.1371/journal.pmed.1001788 25734259 PMC4348198

[32] Pemberton-Ross P, Smith TA, Hodel EM, Kay K, Penny MA. Age-shifting in malaria incidence as a result of induced immunological deficit: a simulation study. Malar J. 2015;14:287 10.1186/s12936-015-0805-1 26206255 PMC4513612

[33] Woolhouse ME. Patterns in parasite epidemiology: the peak shift. Parasitol Today. 1998;14(10):428–34. 17040835 10.1016/s0169-4758(98)01318-0

[34] Phimpraphi W, Paul RE, Yimsamran S, Puangsa-art S, Thanyavanich N, Maneeboonyang W, et al Longitudinal study of *Plasmodium falciparum* and *Plasmodium vivax* in a Karen population in Thailand. Malar J. 2008;7:99 10.1186/1475-2875-7-99 18518964 PMC2443811

[35] Noor AM, Moloney G, Borle M, Fegan GW, Shewchuk T, Snow RW. The use of mosquito nets and the prevalence of *Plasmodium falciparum* infection in rural South Central Somalia. PLoS One. 2008;3(5):e2081 10.1371/journal.pone.0002081 18461178 PMC2362695

[36] Cernetich A, Garver LS, Jedlicka AE, Klein PW, Kumar N, Scott AL, et al Involvement of gonadal steroids and gamma interferon in sex differences in response to blood-stage malaria infection. Infect Immun. 2006;74(6):3190–203. 10.1128/IAI.00008-06 16714546 PMC1479253

[37] Daniels RF, Schaffner SF, Wenger EA, Proctor JL, Chang HH, Wong W, et al Modeling malaria genomics reveals transmission decline and rebound in Senegal. Proc Natl Acad Sci U S A. 2015;112(22):7067–72. 10.1073/pnas.1505691112 25941365 PMC4460456

[38] Baniecki ML, Faust AL, Schaffner SF, Park DJ, Galinsky K, Daniels RF, et al Development of a single nucleotide polymorphism barcode to genotype *Plasmodium vivax* infections. PLoS Negl Trop Dis. 2015;9(3):e0003539 10.1371/journal.pntd.0003539 25781890 PMC4362761

[39] Bei AK, Diouf A, Miura K, Larremore DB, Ribacke U, Tullo G, et al Immune characterization of *Plasmodium falciparum* parasites with a shared genetic signature in a region of decreasing transmission. Infect Immun. 2015;83(1):276–85. 10.1128/IAI.01979-14 25368109 PMC4288878

[40] Liljander A, Chandramohan D, Kweku M, Olsson D, Montgomery SM, Greenwood B, et al Influences of intermittent preventive treatment and persistent multiclonal *Plasmodium falciparum* infections on clinical malaria risk. PLoS One. 2010;5(10):e13649 10.1371/journal.pone.0013649 21048970 PMC2965101

[41] Crompton PD, Miura K, Traore B, Kayentao K, Ongoiba A, Weiss G, et al In vitro growth-inhibitory activity and malaria risk in a cohort study in mali. Infect Immun. 2010;78(2):737–45. 10.1128/IAI.00960-09 19917712 PMC2812204

[42] Calderaro A, Piccolo G, Perandin F, Gorrini C, Peruzzi S, Zuelli C, et al Genetic polymorphisms influence *Plasmodium ovale* PCR detection accuracy. J Clin Microbiol. 2007;45(5):1624–7. 10.1128/JCM.02316-06 17360843 PMC1865880

