# Supplementary material for: High Plasmodium falciparum longitudinal prevalence is associated with high multiclonality and reduced clinical malaria risk in a seasonal transmission area of Mali
Source: PLoS One. 2017 Feb 3;12(2):e0170948. doi: 10.1371/journal.pone.0170948 (PMC5291380; doi:10.1371/journal.pone.0170948)
Supplement: S1 Fig — At all 4 time-points (A-D), individuals with high COI also had high PmP (Kruskal-Wallis test, all P<0.001). (PDF) [file pone.0170948.s001.pdf]

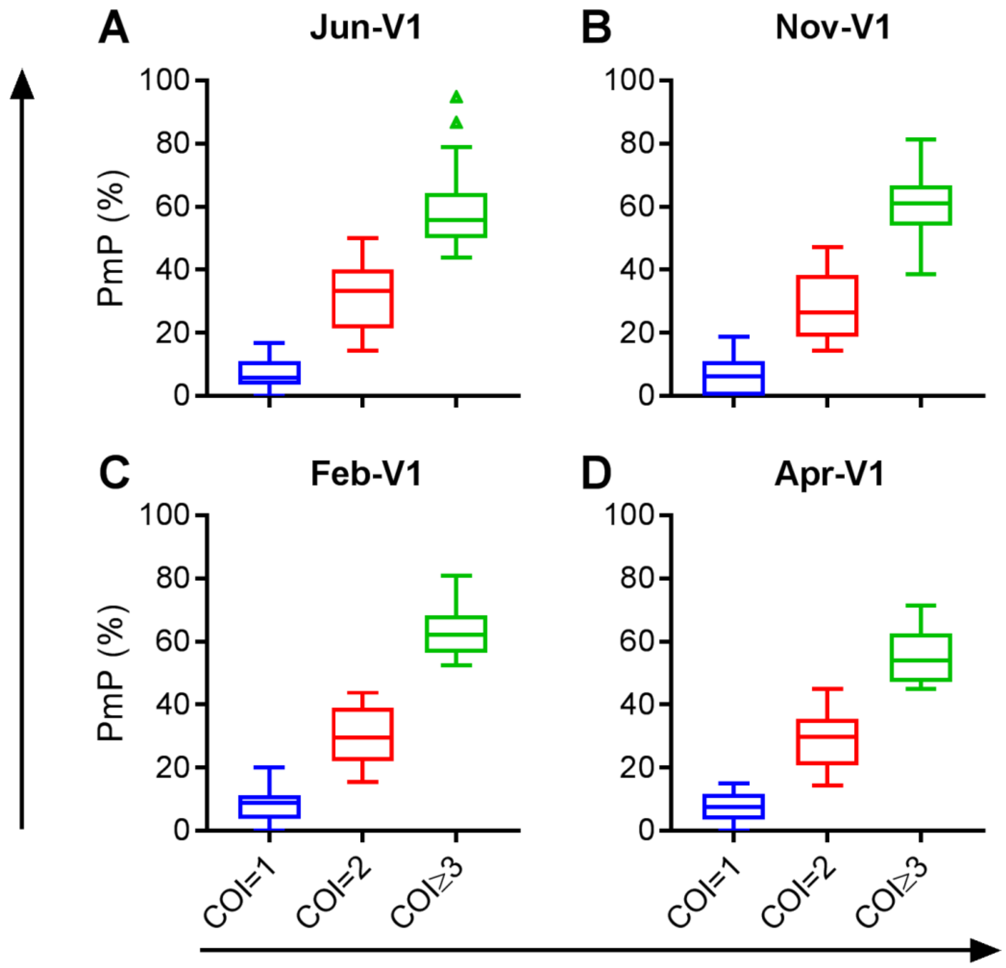

S1 Fig. Distribution of polymorphic proportion (PmP) values among 3 COIL-estimated complexity of infection (COI) levels. At all 4 time-points (*A*-*D*), individuals with high COI also had high PmP (Kruskal-Wallis test, all $P<0.001$).
