# Supplementary material for: High Plasmodium falciparum longitudinal prevalence is associated with high multiclonality and reduced clinical malaria risk in a seasonal transmission area of Mali
Source: PLoS One. 2017 Feb 3;12(2):e0170948. doi: 10.1371/journal.pone.0170948 (PMC5291380; doi:10.1371/journal.pone.0170948)
Supplement: S1 Table — (DOCX) [file pone.0170948.s002.docx]

**Table S1. Nested PCRs to Detect *P. falciparum* in Dried Blood Spots**

| **Reagent** | **Volume (20-µL total)** | **Final concentration** |
| --- | --- | --- |
| ***Nest 1*** |  |  |
| DNA template | 1-mm circular punch of dried blood spot |  |
| 2X Phusion Blood PCR Buffer | 10 µL | 1X |
| rPLU5 | 0.08 µL | 0.4 µM |
| rPLU6 | 0.08 µL | 0.4 µM |
| Phusion Blood II DNA Polymerase | 0.40 µL |  |
| H_2_O | 9.44 µL |  |
| ***Nest 2*** | | |
| Nest 1 Reaction | 1 µL |  |
| 2X GoTaq Master Mix | 10 µL | 1X |
| rFAL1^a^ | 0.08 µL | 0.4 µM |
| rFAL2^a^ | 0.08 µL | 0.4 µM |
| H_2_O | 8.84 µL |  |

^a^ Species-specific primer sets were used for secondary reactions. *P. malariae* infection was tested with rMAL1 and rMAL2 primer set (Snounou et al., 1993).
