# Supplementary material for: High Plasmodium falciparum longitudinal prevalence is associated with high multiclonality and reduced clinical malaria risk in a seasonal transmission area of Mali
Source: PLoS One. 2017 Feb 3;12(2):e0170948. doi: 10.1371/journal.pone.0170948 (PMC5291380; doi:10.1371/journal.pone.0170948)
Supplement: S2 Table — (DOCX) [file pone.0170948.s003.docx]

**Table S2: Thermocycler Profiles of Nested PCRs to Detect *P. falciparum* in Dried Blood Spots**

| **Step** | **Temperature** | **Time** | **Cycle** |
| --- | --- | --- | --- |
| ***Nest 1*** |  |  |  |
| Cell lysis and polymerase activation | 98^o^C | 5 min | 1X |
| Denaturation | 98^o^C | 5 sec | 30X |
| Annealing | 58^o^C | 5 sec |  |
| Extension | 72^o^C | 30 sec |  |
| Final extension | 72^o^C | 5 min | 1X |
| ***Nest 2*** | | | |
| Initial denaturation | 95^o^C | 3 min | 1X |
| Denaturation | 95^o^C | 30 sec | 35X |
| Annealing | 60^o^C | 30 sec |  |
| Extension | 72^o^C | 30 sec |  |
| Final extension | 72^o^C | 30 sec | 1X |
