# Supplementary material for: High Plasmodium falciparum longitudinal prevalence is associated with high multiclonality and reduced clinical malaria risk in a seasonal transmission area of Mali
Source: PLoS One. 2017 Feb 3;12(2):e0170948. doi: 10.1371/journal.pone.0170948 (PMC5291380; doi:10.1371/journal.pone.0170948)
Supplement: S4 Table — (DOCX) [file pone.0170948.s005.docx]

| **Years** | **1-2** | **3-4** | **5-6** | **7-8** | **9-10** | **11-12** | **13-16** | **17-30** | **31-40** |
| --- | --- | --- | --- | --- | --- | --- | --- | --- | --- |
| **3-4** | >0.9999 |  |  |  |  |  |  |  |  |
| **5-6** | >0.9999 | 0.1628 |  |  |  |  |  |  |  |
| **7-8** | >0.9999 | **0.0207** | >0.9999 |  |  |  |  |  |  |
| **9-10** | **<0.0001** | **<0.0001** | 0.0519 | 0.152 |  |  |  |  |  |
| **11-12** | **0.0024** | **<0.0001** | 0.5794 | >0.9999 | >0.9999 |  |  |  |  |
| **13-16** | **0.0001** | **<0.0001** | 0.054 | 0.1485 | >0.9999 | >0.9999 |  |  |  |
| **17-30** | 0.9999 | >0.9999 | >0.9999 | >0.9999 | **0.0029** | **0.0482** | **0.0036** |  |  |
| **31-40** | 0.9999 | >0.9999 | 0.7827 | 0.1758 | **<0.0001** | **0.0001** | **<0.0001** | >0.9999 |  |
| **>40** | 0.9477 | >0.9999 | **0.0015** | **0.0001** | **<0.0001** | **<0.0001** | **<0.0001** | 0.144 | >0.9999 |

**Table S4.** Adjusted *P* Values* from Dunn’s Multiple Comparison Test for Age-stratified *P. falciparum* Longitudinal Prevalence (PfLP) Data

* *P*<0.0001 by Kruskal-Wallis test when all age groups were compared
